# Genealogy and clinical course of catecholaminergic polymorphic ventricular tachycardia caused by the ryanodine receptor type 2 P2328S mutation

**DOI:** 10.1371/journal.pone.0243649

**Published:** 2020-12-14

**Authors:** Mikael Koponen, Annukka Marjamaa, Annukka M. Tuiskula, Matti Viitasalo, Terhi Nallinmaa-Luoto, Jaakko T. Leinonen, Elisabeth Widen, Lauri Toivonen, Kimmo Kontula, Heikki Swan

**Affiliations:** 1 Heart and Lung Center, Helsinki University Hospital, Helsinki, Finland; 2 University of Helsinki, Helsinki, Finland; 3 Department of Medicine, Helsinki University Hospital and University of Helsinki, Helsinki, Finland; 4 Laboratory of Genetics, HUSLAB, Helsinki University Hospital, Helsinki, Finland; 5 University of Turku, Turku, Finland; 6 Institute for Molecular Medicine Finland, FIMM, University of Helsinki, Helsinki, Finland; Ohio State University, UNITED STATES

## Abstract

**Background:**

Catecholaminergic polymorphic ventricular tachycardia (CPVT) is a severe inherited arrhythmic disease associated with a risk of syncope and sudden cardiac death (SCD).

**Aims:**

We aimed at identifying *RYR2* P2328S founder mutation carriers and describing the clinical course associated with the mutation.

**Methods:**

The study population was drawn from the Finnish Inherited Cardiac Disorder Research Registry, and from the present genealogical study. Kaplan-Meier graphs, log-rank test and Cox regression model were used to evaluate the clinical course.

**Results:**

Genealogical study revealed a common ancestor couple living in the late 17^th^ century. A total of 1837 living descendants were tested for *RYR2* P2328S mutation unveiling 62 mutation carriers aged mean 39±23 years old. No arrhythmic deaths were documented among genotyped subjects, but 11 SCDs were detected in non-genotyped family members since 1970. Three genotyped patients (5%) suffered an aborted cardiac arrest (ACA), and 15 (25%) had a syncope triggered by exercise or stress. Rate of cardiac events was higher among patients who in exercise stress test showed a maximum rate of premature ventricular contractions >30/min (68% vs 17%, p<0.01; hazard ratio = 7.1, p = 0.02), in comparison to patients without the respective finding. A cardioverter-defibrillator (ICD) was implanted to 13 (22%) patients, with an appropriate ICD shock in four (31%) subjects. All ICD shocks, one ACA, and one syncope occurred during β-blocker medication.

**Conclusions:**

Previously undiagnosed CPVT patients may be identified by well-conducted genealogical studies. The *RYR2* P2328S mutation causes a potentially severe phenotype, but its expression is variable, thus calling for additional studies on modifying factors.

## Introduction

Catecholaminergic polymorphic ventricular tachycardia (CPVT) is a severe inherited arrhythmic disease in patients with structurally normal heart and normal resting electrocardiogram (ECG) [1–3]. Affected individuals present with bidirectional or polymorphic ventricular tachycardia typically during exercise or emotional stress predisposing to syncope and sudden cardiac death (SCD) [4–6]. CPVT results from disruptions in calcium ion homeostasis in cardiac myocytes. The most common form of CPVT, representing about two-thirds of cases, is caused by gain-of-function defects of the ryanodine receptor type 2 (*RYR2*) calcium channel present in sarcoplasmic reticulum [7–9]. Knowledge concerning risk factors of cardiac events is incomplete [10–13]. β-adrenergic blockers are the first-line treatment in CPVT, and in selected cases flecainide is combined with β-blockers [10, 12, 14–16]. Implantable cardioverter-defibrillator (ICD) has been recommended to patients who suffer an aborted cardiac arrest (ACA), or remain symptomatic despite optimal drug therapy [15, 17].

The Finnish founder mutation *RYR2* P2328S identified in 2001 was one of the first described CPVT-causing mutations [8]. Its pathophysiological properties have been characterized in detail by in vitro studies [18]. In this paper, we describe the identification of an apparent common ancestor couple with this founder mutation, living in Central Finland in the 17^th^ and 18^th^ century, and use the resultant large family tree to identify additional CPVT patients with the same mutation. In addition, we describe relevant data on the clinical course associated with a carrier status of the *RYR2* P2328S mutation.

## Materials and methods

### Genealogical study and study population

The study population was drawn partly from the Finnish Inherited Arrhythmic Disorder Research Registry established in 1991, and partly from the present genealogical study. Probands of apparently unrelated families were diagnosed with *RYR2* P2328S mutation. Old Finnish parish records were used to construct family trees with merging pattern backwards up to the late 17^th^ century. Following the identification of the proposed ancestor couple, it was possible to construct a pedigree back to the presently living individuals. After retrieving their addresses from The Finnish Population Data Services Agency, they were contacted with a letter and invited to donate a blood sample for DNA analysis. Of the 2245 contacted, 1837 (82%) agreed to participate.

Individuals tested positive for *RYR2* P2328S mutation were included in the exercise stress test (EST) and clinical course analyses. The follow-up time started from birth and ended 1) at last follow-up, or 2) when patient deceased. Data of all deaths during the follow-up were obtained from Statistics Finland by means of social security number search. Medical records of all genotyped mutation carriers were obtained. The decision whether to initiate β-blocker or flecainide therapy, or to implant ICD was made by the responsible physician without consulting the authors. The study was approved by the Ethical Review Committee of Helsinki University Hospital, and a written informed consent was obtained from the study subjects. The Ministry of Social Affairs and Health consented for the participation of deceased subjects. All study subjects were of Finnish origin.

### Analysis of the *RYR2* P2328S mutation and haplotype analysis

The *RYR2* (NM_001035.2) c.6982C>T p.(Pro2328Ser) mutation was detected in immediate family members of the probands using a mutation-specific restriction enzyme assay or direct DNA sequencing, as described by Kujala et al. [19]. From participants of the genealogical study, DNA was extracted by submerging a blank strip (MQuant™, Merck, Darmstadt, Germany) into EDTA blood as described previously [20]. Strips were dried and incubated overnight in 70°C submerged in 1x PCR buffer (AmpliTaq Gold™ 10x PCR Buffer containing MgCl2, Thermo Fisher Scientific, Waltham, MA, USA). The tubes were centrifuged and the supernatant was used as a template in PCR (forward primer: tgc aag caa aat tta ctg tgt ctc, reverse primer: ttc cag cac caa att cca tt). The PCR products were directly sequenced using BigDye™ Terminator v3.1 Cycle Sequencing Kit and ABI 3730xl DNA Analyzer (Thermo Fisher Scientific). For haplotype analysis, a subset of the probands and carriers of the *RYR2* P2328S mutation representing distant relatives were subjected to genotyping by HumanOmniExpress chip (Illumina, San Diego, CA USA) at the FIMM technology centre. Haplotypes for the probands and the frequency of the carrier haplotype in a Finnish reference cohort (GeneRisk, www.generisk.fi) containing 7580 subjects were determined using PLINK 1.07 [21].

### Statistical analyses

Clinical characteristics were analyzed using Fisher’s exact test for categorical, and Wilcoxon rank-sum test or unpaired t-test for continuous variables. The definition of a cardiac event was CPVT-related syncope, ACA, appropriate ICD shock, or SCD. A syncope triggered by physical or emotional stress was defined as CPVT-related syncope, and a resuscitation requiring external defibrillation was defined as ACA. A death was regarded as being SCD if the event was abrupt in onset without evident cause if witnessed, or was not explained by any other cause if it occurred in an unwitnessed setting. The definition of ventricular tachycardia (VT) was at least three consecutive bidirectional or polymorphic premature ventricular contractions (PVC). Bigeminy, couplet, or VT in EST were regarded as typical arrhythmias of CPVT. The maximum rate of PVCs /min in EST was counted manually, and irrespective whether bigeminy, couplet, or VT was present. The end point for statistical analyses was each subject’s first cardiac event. Kaplan-Meier methods were utilized to depict the cumulative incidence rate (= cumulative probability) of first cardiac event. The significance of the differences was tested by the log-rank test. Multivariate Cox proportional hazards regression model was used to evaluate the independent contribution of clinical risk factors. The findings of each individual’s first EST off β-blocker medication were used in risk factor investigation. No violation of the proportional hazards assumption was detected as tested by log-log graphs, and no statistically significant interactions were discovered in interaction term analyses. Analyses were carried out using SPSS version 22 or newer. A 2-sided p-value ≤0.05 was interpreted as statistically significant.

## Results

### Genealogical study

**Fig 1:**
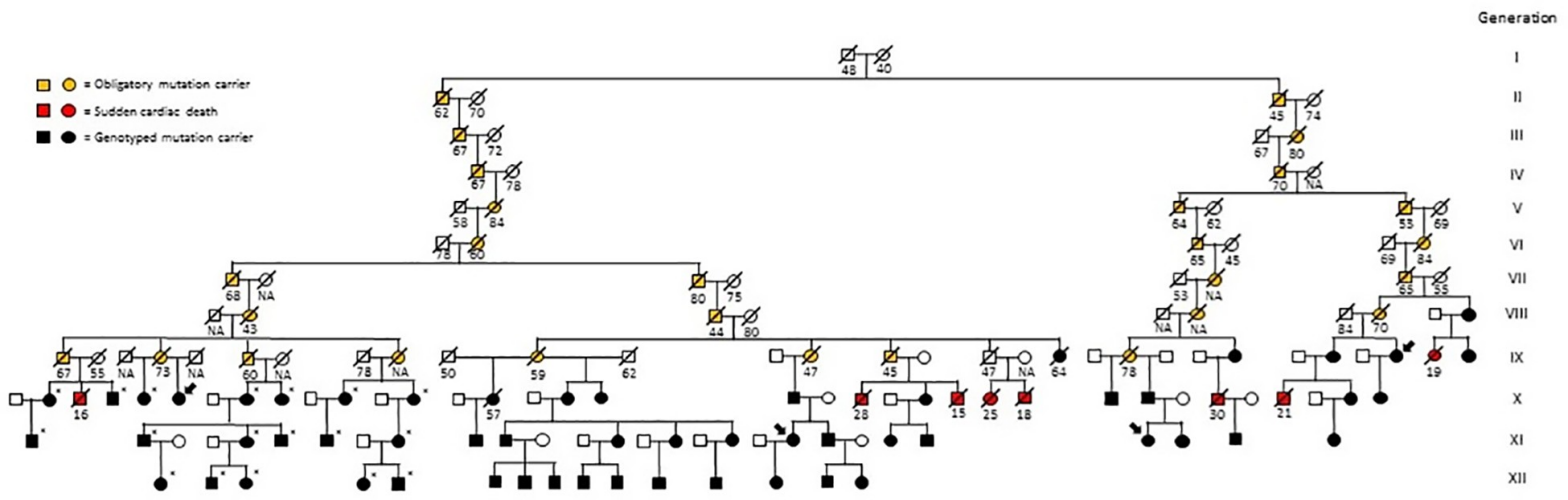
The family tree showing the common ancestors born in 1670 and 1677, mutation carriers, and family members who suffered a sudden cardiac death (SCD) in 1970 or later. Siblings of obligatory mutation carriers and subjects in which the mutation was ruled out by genetic testing are not shown. All SCDs occurred before the era of genetic testing. In five genotyped and presently living mutation carriers (two probands and three relatives), and in their three non-genotyped family members with SCD (one obligatory mutation carrier and two relatives), the identification of a linkage to the presented family tree was unsuccessful. Arrows indicate probands, asterisks indicate subjects who were diagnosed based on the current genealogical study, and numbers below individuals denote the age at death. NA = not available.

Since the initial detection of the *RYR2* P2328S mutation in a large Finnish family with CPVT, [7, 8] we have identified three other families with the same mutation; the pedigrees of these families appear in the composite family tree shown in Fig 1. Due to the isolated nature of the Finnish population, a suspicion of kinship arouse and was strengthened by demonstration of the subjects sharing a haplotype within the *RYR2* gene region. Genealogical studies, starting with the probands and utilizing the Finnish parish registers, revealed a putative common ancestor couple who were born in 1670 and 1677 (Fig 1). They lived in the Viitasaari parish, Central Finland, and there have been up to 11 generations of descendants since. All generations included, the age of death was similar in obligatory mutation carriers compared to their spouses (mean 63.1 and 67.3 years, respectively, p = 0.26). Between generations II-V (n = 9) and VI-X (n = 19) the age of death in obligatory mutation carriers was similar (66 vs 61 years, respectively, p = 0.39). The mean number of children per obligatory mutation carrier was 5.9. A total of 1837 living descendants of the common ancestors were tested for *RYR2* P2328S mutation. Of them, 62 were carriers of the mutation, and 1775 were non-carriers. The genealogical study revealed 18 previously undiagnosed mutation carriers. Many branches of the total family tree (not shown) had no mutation carriers, whereas a few branches had clusters of mutation carriers.

Investigations regarding non-genotyped relatives who died in 1970 or later showed two SCDs in obligatory mutation carriers, and additional nine SCDs among relatives who were suspected to have carried the mutation (Fig 1). Of the family members suffering SCD, seven were males and four were females. In eleven subjects, including two obligatory mutation carriers, data regarding the cause of death were insufficient. Four of the SCDs in non-genotyped family members were reported in our previous study [7].

### Clinical course and risk factors for cardiac events

Of the genotyped mutation carriers three were not taking part to the study, while 59 subjects were eligible to clinical course analyses. The mean total follow-up time, including retrospectively collected data from birth on, was 38±24 years.

The clinical characteristics of the genotyped mutation carriers are summarized in Table 1. Neither of the two deaths were arrhythmia-related. Three patients (5%) suffered an ACA, and 15 (25%) had a CPVT-related syncope. The first cardiac event occurred at the age of 21.9 and 12.6 in females and males, respectively (p = 0.12). At diagnosis 15 (25%) of the subjects were symptomatic. The cumulative rate of cardiac events was similar between females and males (45% vs 38%, respectively, p = 0.71), and regardless of whether the mutation had been inherited from mother or father (46% vs 42%, respectively, p = 0.54). Family history of cardiac events in first degree relatives associated with a higher rate of events (53% vs 0%, p = 0.04).

**Table 1:** Characteristics of *RYR2* P2328S mutation carriers by symptom status.

	No CE n = 41 (69%)	CE* n = 18 (31%)	P-value†
Female, n (%)	24 (59)	13 (72)	0.39
Proband, n (%)	1 (2)	5 (28)	<0.01
Age at follow-up end, y, mean±SD	33.3±24.5	49.9±16.4	<0.01
Prospective follow-up, y, mean±SD	7.4±8.1	11.4±7.0	0.05
Age at baseline ECG, y, mean±SD‡	21.9±20.1	31.8±18.2	0.11
Heart rate, 1/min, mean±SD	80.4±15.3	66.3±10.3	<0.01 ††
Heart rate (age >10y), 1/min, mean±SD	71.8±9.6	65.6±10.5	0.13 ††
QTc, ms, mean±SD	397±22	403±20	0.41 ††
Family history of CE, n (%)§	19 (51)	15 (100)	<0.01
β-blocker, n (%)	38 (93)	18 (100)	0.55
Age at starting, y, mean±SD	26.5±23.4	35.6±19.8	0.09
Follow-up time with, y, mean±SD	7.0±8.0	14.3±11.2	<0.01
Side effects, n (%)	7 (18)	6 (33)	0.31
ICD, n (%)	2 (5)	11 (61)	<0.01
Appropriate shock, n (%)	0	4 (36)	1.00
Inappropriate shock, n (%)	1 (50)	1 (9)	0.30
Age at implantation, y, mean±SD	21.9±7.2	36.9±13.8	0.23
Follow-up time, y, mean±SD	6.8±7.5	9.2±5.7	1.00
Complication, n (%)	1 (50)	2 (18)	0.42
Revision, n (%)| |	1 (50)	8 (73)	1.00
EST, n (%)	31 (76)	17 (94)	0.15
Without β-blocker, n (%)	23 (74)	14 (82)	0.72
PVC, n (%)#	15 (65)	13 (93)	0.11
Bigeminy, n (%)#	11 (48)	13 (93)	0.01
Couplet, n (%)#	9 (39)	10 (71)	0.09
VT, n (%)#**	6 (26)	7 (50)	0.17
Max PVC rate >30/min#	8 (35)	12 (86)	<0.01

* CPVT-related syncope, ACA, appropriate ICD shock, or SCD.

† P-value ≤0.05 regarded as statistically significant.

‡ Only ECGs with sinus rhythm, and off β-blocker and flecainide medication included (n = 38). Two patients had atrial fibrillation, and one had atrioventricular sequential paced rhythm in their ECG.

§ In first degree relatives.

| | Generator replacements due to battery depletion not included.

# In the first EST without β-blocker medication.

** Bidirectional or polymorphic.

†† Normally distributed. Unpaired t-test used for testing.

CE = cardiac event, ECG = electrocardiogram, EST = exercise stress test, ICD = implantable cardioverter-defibrillator, SD = standard deviation, VT = ventricular tachycardia.

The number of probands in the study was six, and they were all females. Probands were more often symptomatic compared to non-probands (83% vs 25%, p<0.01). Of the probands two (33%) suffered an ACA, and one (25%) had an appropriate ICD shock therapy. ICD was implanted to four (67%) probands.

**Fig 2:**
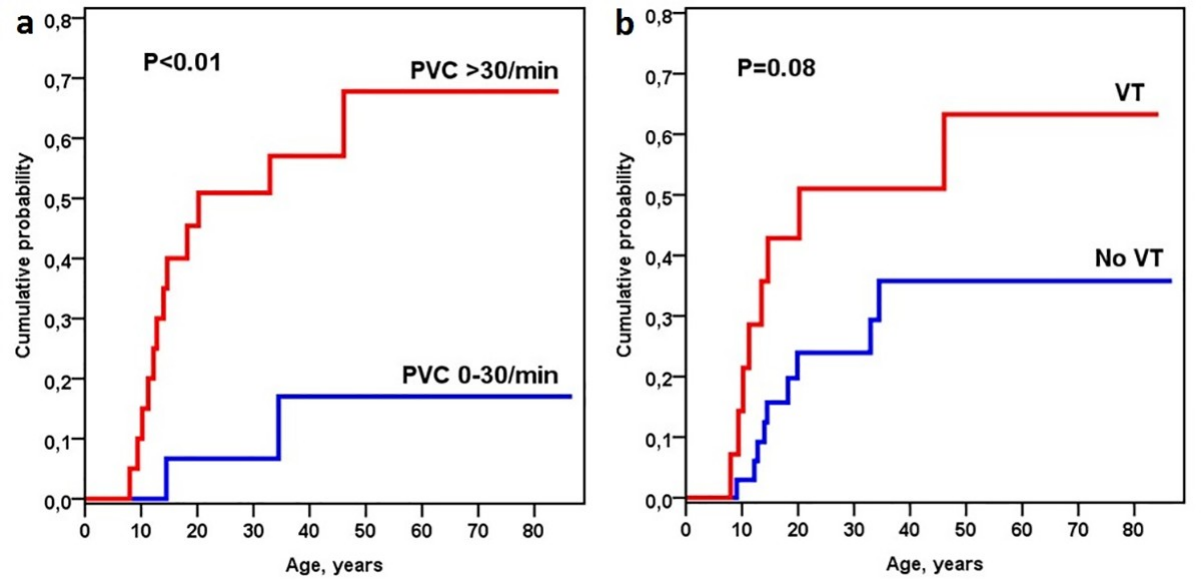
Exercise stress test (EST) findings of each individuals first EST off β-blocker medication (n = 37) and cumulative probability of cardiac events by (a) maximum rate of premature ventricular contractions [PVC], and (b) ventricular tachycardia [VT].

At least one exercise stress test (EST) was carried out in 48 (81%) subjects, and the mean number of ESTs was 3.9 (range 1–12). Of the subjects, 37 (77%) had an EST off β-blocker medication. Using the results of ESTs, risk factors for cardiac events were analyzed. Rate of cardiac events was higher in patients who in the first EST off β-blocker showed a maximum rate of PVCs >30/min (68% vs 17%, p<0.01, Fig 2A; hazard ratio = 7.1, p = 0.02, Table 2), or bigeminy (61% vs 17%, p = 0.02) in comparison to patients without the respective finding. Occurrence of VT (p = 0.21, Fig 2B), PVC threshold heart rate (<120 vs ≥120 /min, p = 0.39), number of PVC forms (1 vs ≥2 forms, p = 0.81), or maximal work capacity (Wmax/weight <2 vs ≥2 W/kg, p = 0.90) in EST were not associated with rate of cardiac events.

**Table 2:** Time-dependent Cox regression model: Risk factors of cardiac events.

	Hazard ratio	95% confidence interval	P-value
Max PVC rate >30/min*	7.13	1.31–38.8	0.02
Male vs female	1.51	0.41–5.59	0.53
β-blocker vs no β-blocker	0.89	0.16–4.90	0.89

β-blocker medication is evaluated in a time-dependent manner.

* ESTs off β-blocker medication (n = 37) are included in the model.

EST = exercise stress test, PVC = premature ventricular contraction.

### Pharmacological treatment

A total of 56 (95%) patients were on β-blocker medication with a mean initiation age of 29.3 years. Time-dependent β-blocker treatment did not have significant impact on risk (β-blocker vs no β-blocker HR = 0.89, p = 0.89) in our cohort (Table 2). The incidence rate of cardiac events per 1000 person-years was 7.5 and 13.3 with and without β-blocker treatment, respectively. Non-selective and selective β-blockers were also tested separately in univariate Cox models (HR = 0.33, p = 0.28; HR = 0.99, p = 0.99 for non-selective and selective, respectively). During appropriate β-blocker treatment four (7%) breakthrough cardiac events occurred (three ICD shocks and one ACA). Of the breakthrough events two occurred during non-selective (propranolol) and two during selective (bisoprolol or metoprolol) β-blocker treatment (7% and 8%, respectively, p = 1.00). β-blocker medication by generic names is presented in the S1 Table.

At the end of follow-up, one male used flecainide combined with β-blocker, and one male had flecainide monotherapy. Three females had discontinued flecainide, and in two cases the reason for this were drug-related side effects. In one subject the indication for flecainide treatment was paroxysmal atrial fibrillation (AF), and when AF progressed to permanent AF, flecainide was discontinued. All patients who discontinued flecainide continued with β-blocker medication. No arrhythmic events occurred during flecainide therapy.

### Device therapy

ICD was implanted to 13 (22%) subjects at a mean age of 34.6±13.9 years, and the mean follow-up time with the device was 8.8±5.7 years. The indications for ICD were ACA (n = 3), syncope during sport (n = 6), and abundant PVCs in EST (n = 4). Additionally, substantial family history of cardiac events was a contributing factor for ICD in several cases. An appropriate ICD shock therapy occurred in four (31%), and an inappropriate shock in two (15%) patients. One subject had not taken β-blocker medication on the day of the defibrillation therapy. The first EST showed typical arrhythmias of CPVT in all patients who suffered an ICD shock: two subjects had VT runs of 5–6 PVCs, one had plentiful PVCs with eight couplets, and one had abundant PVCs with bigeminy. During the appropriate ICD shock two subjects had a ventricular fibrillation (VF) that was preceded by VT, one subject had VF that was preceded by PVCs but not VT, and one subject had VT. Complications were encountered in three (23%) subjects (incidence rate 26 per 1000 person-years): a lead damage causing an inappropriate shock (n = 1), a lead displacement (n = 1), and an inappropriate shock during atrial tachycardia (n = 1). Two complications necessitated revision (15%). Pacemaker was implanted to three (5%) patients at a mean age of 76.8±7.9, and implantation indication in all cases was sick sinus syndrome.

## Discussion

### The *RYR2* P2328S mutation: past and present

We here report the probable origin of the *RYR2* P2328S mutation, taking advantage of the postulated distant relatedness of four alive probands, as well as relevant clinical characteristics associated with a carrier status of this mutation. The genealogical study revealed 18 new living mutation carriers, underscoring the importance of family screening to identify patients at risk of cardiac events. The relatively small portion of molecularly confirmed mutation carriers out of the tested subjects (62/1837) is not unexpected since at every generation, the risk of inheriting the mutation decreases. Our DNA analyses included subjects mostly from the 10^th^-12^th^ generations (Fig 1).

Previously, RYR2 channel with P2328S mutation was shown to have a leaky Ca2+ release gain-of-function defect under sympathetic activation in vitro [18, 22]. Expression studies indicated non-alternating variability of Ca2+ transients in response to adrenergic agent stimulation generating delayed afterdepolarizations which in turn may trigger ventricular tachyarrhythmia [19, 23, 24]. Further, repolarization abnormalities as a form of U-wave to T-wave ratio, and short-term variability of QT interval have been demonstrated in vivo [23, 25]. *RYR2* P2328S mutation has also been associated with atrial arrhythmogenic properties [26].

The current study complements the previous studies by providing a more detailed description of clinical course on top of the previous insights of molecular mechanisms and clinical electrocardiography findings. The initial description of the *RYR2* P2328S mutation favored its highly malignant nature [7, 8]. The present study, however, indicates that the associated phenotype is more variable which may reflect incomplete penetrance in occasional cases. It should be emphasized that the statistical analyses of the current study includes only subjects tested positive for the mutation, omitting the SCD cases in which DNA analyses and clinical investigations were missing. The management and clinical course of the subjects without DNA analyses could have been different if the genotype status would have been known. We also noticed the lifespan was rather similar among the obligatory mutation carriers and their spouses, which could indicate selection due to milder phenotype in these mutation carriers who reached sexual maturity. Collectively, our previous and present studies thus indicate that the phenotype associated with the *RYR2* P2328S mutation is severe enough to necessitate prompt management.

Recently, a similar study described a CPVT founder mutation population affected by the *RYR2* G357S mutation, showing disease penetrance and expression reminiscent to those of our study [27]. Also, a single EST was often insufficient to detect typical cardiac arrhythmias of CPVT, though serial stress tests improved accuracy [27]. Indeed, the observed variable expression of the specific CPVT-causing mutations calls for further investigation on additional genetic and environmental factors.

### Risk factors for cardiac events

Possibilities for risk stratification in CPVT are limited. We present that maximum rate of PVCs >30/min in EST is associated with a higher risk of cardiac events. In accordance with previous studies, bigeminy was also associated with a higher risk [10, 11, 13]. However, also subjects with a normal EST have shown cardiac events during follow-up [10, 11]. Indeed, in a previous study couplets or more consecutive PVCs in EST predicted future cardiac events with a modest sensitivity and specificity of 62% and 67%, respectively [10]. In accordance with previous studies, probands had a higher cardiac event rate compared to their affected family members [13, 28]. Similar to the most of the previous studies, our study indicated a similar risk of cardiac events in both genders [10, 28].

### Pharmacological treatment

According to the recommendations of the 2015 ESC Guidelines, all patients with a diagnosis of CPVT should avoid competitive sports, strenuous exercise and stressful environments. Administration of β-blockers are recommended to all patients with a clinical diagnosis of CPVT, and also to genetically positive family members even after negative EST [15]. Due to lack of deceased genotyped subjects and relatively short follow-up time with β-blockers, we were unable to assess the effect of β-blocker treatment on survival. Breakthrough events took place equally often during administration of selective vs. non-selective β-blockers, while in some previous studies non-selective compared to selective β-blockers have been more effective in preventing arrhythmias [10, 29, 30]. Which ever type of β-blocker is used, it is important that drug treatment continues uninterrupted at highest tolerated dose [10, 15, 28].

### Device therapy

In the present study, the rate of appropriate ICD shocks was similar, but the rate of inappropriate shocks and complications appeared lower compared to earlier studies [31, 32]. Previously, recurrent inappropriate ICD shocks in CPVT patients have been encountered predisposing to electrical and catecholaminergic storm, a detrimental condition in CPVT patients [28, 31, 33]. Also, in a recent study previously undiagnosed patients with CPVT who presented with an ACA, implantation of an ICD was not associated with improved survival [32]. These observations emphasize careful assessment of device implantation in CPVT patients, and the importance of strict adherence to pharmacological therapy [15, 31, 32].

### Study limitations

Our study comprises carriers of a specific *RYR2* mutation which needs to be taken into account when attempting to generalize the results to other CPVT subjects. Most of the follow-up was assessed in retrospective manner which may cause recall bias. The statistical analyses of clinical course included only genotyped mutation carriers. Thus, the exclusion of the SCD cases in non-genotyped family members, and the limited sample size precluded assessment of the effect of β-blocker treatment on survival. In five genotyped mutation carriers identification of a genealogical linkage to the presented family tree was unsuccessful.

## Conclusions

This study, presenting a relatively large genetically homogenous sample of CPVT patients, underscores the importance of molecular screening to identify family members at risk of cardiac events. The *RYR2* P2328S mutation causes a potentially severe phenotype, but its expression is variable, thus calling for additional studies on modifying factors. Although risk stratification remains challenging, EST findings may be of help in risk assessment.

## Supporting information

S1 Table β-blockers by generic names. (DOCX)

S2 Table Anonymized data set. (XLSX)
