# Supplementary material for: Genealogy and clinical course of catecholaminergic polymorphic ventricular tachycardia caused by the ryanodine receptor type 2 P2328S mutation
Source: PLoS One. 2020 Dec 14;15(12):e0243649. doi: 10.1371/journal.pone.0243649 (PMC7735638; doi:10.1371/journal.pone.0243649)
Supplement: S1 Table — (DOCX) [file pone.0243649.s001.docx]

**S1 Table.**

|  | Propranolol  (n=28, 50%) | Bisoprolol  (n=21, 38%) | Atenolol  (n=4, 7%) | Metoprolol  (n=3, 5%) | P-value |
| --- | --- | --- | --- | --- | --- |
| Dose, mg/kg/d, mean±SD | 2.3±1.1 | 0.13±0.07 | 1.1±0.3 | 0.9±0.6 | NA |
| Follow-up with, y, mean±SD | 10.3±11.6 | 6.5±5.1 | 14.0±10.1 | 17.1±12.6 | 0.28 |
| Cardiac event during, n (%) | 2 (7) | 1 (5) | 0 | 1 (33) | 0.41 |
| Side effects, n (%) | 7 (25) | 4 (19) | 0 | 1 (33) | 0.84 |
